# Eyestrain and Crime

**Published:** 1906-10

**Authors:** George M. Gould

**Affiliations:** Philadelphia


### Eyestrain and Crime.

BY GEORGE M. GOULD, M. D., Philadelphia.

BY the kindness of Dr. Geo. M. Case of Elmira, New York, Ophthalmologist of the Elmira State Reformatory, I epitomise from his paper read before the American Academy of Ophthalmology and Oto-Laryngology, August 30-31, and September 1, 1906, the following facts. The calculations are made from the diagnoses of the refraction of 400 inmates chosen as illustrative of less than 5000 patients examined during the years of his service. The report relates to inmates with:

| | | |
|---|---|---|
| Hyperopia, simple | 68 | |
| Compound hyperopic atsigmatism | 27 | |
| Hyperopic astigmatism | 37 | |
| With Predominant Hyperopia | | 132 |
| Myopia, simple | 37 | |
| Compound myopic astigmatism | 19 | |
| Myopic astigmatism | 18 | |
| With Predominant Myopia | | 74 |
| Mixed astigmatism | | 7 |
| Strabismus | | 10 |
| Total with Ametropia and Strabismus | | 223 |
| Of these the errors were corrected in | | 146 |
| Uncorrected | | 77 |
| | | 223 |

It is noteworthy that of these errors an astonishing proportion had very high degrees of ametropia, the defect in 53 cases being from 2 D. to 4 D., and in 37 cases over 4 D. In 10 cases the astigmatism was over 4 D.

It must be remembered that the rules governing these tests were such that in some ways make the results untrustworthy, but that all the conditions imposed by the State demonstrate that only the most glaring defects were brought out. As a rule no mydriaic could be used, so that these are only the manifest errors. Anisometropia and heterophia were ordered to be ignored! Then the sole aim permitted was to give approximately normal vision in one eye only. It is of course well known by Dr. Case and by all competent oculists, that the ametropia which produces the most ruinous systemic reflexes is precisely that covered up by the accommodation and permitting in one or both eyes a fair or even good acuteness of 20-foot vision. This fact throws an astonishing and appalling light upon the handicap of these young criminals in attempting almost any work of civilization. If in 90 patients out of 400 the manifest errors were over 2 D., and if the manifest astigmatisms were 108, (45 over 2 D.) the argument is convincing that instead of punishing these afflicted young men, they should be rewarded by giving them accurate and scientific glasses, to enable them to make their living. The State in its infinite stupidity doubly punishes them both by imprisonment and by improper glasses. Such is governmental wisdom.

Put upon 108 out of any 400 moral and well-raised boys of 12 years of age, spectacles which would correct the ametropia of any one of these 108 boys of the reformatory, and they will either get into the reformatory, a hospital, or their graves, within a few years. Let the legislators or officers of the government read or work at any handicraft for one day with such glasses (creating the defects these boys have by nature) and there would be different laws enacted, at the next legislative sessions, and the administrators would see that they were executed! Have societies of learned ophthalmologists, have general medical societies, have penologists, asked for, or demanded laws and appropriations to give these abused boys vision and normal visual function to enable them to do work and live moral lives? The State will not only not pay the oculist, it will not let him do his work scientifically, it will not buy the glasses, it will not care to have the glasses fitted by an expert optician. What amazing blundering! It would be a money-making business if a private oculist and optician would take the job of reducing the State appropriations for criminal institutions 50 or 75 percent, easily done with preventing the ocular diseases and reflexes which directly cause crime in the young. Write ten hours with any compound hyperopic astigmatic spectacles of one, two, or three diopter strength, and be convinced!

What does the State do? Listen again to Dr. Case: He sent letters of inquiry to 123 penal institutions asking if visual acuteness was tested when the boy or prisoner was received; what were the results; were glasses prescribed: the effect on conduct, etc.; were oculists employed; were appropriations made for such work, and the like. Facts were so profuse, interest in the subject was so intense, and conviction so manifest, that no answers were made by 63 institutions! "No facts to report," is probably the most charitable explanation. Dr. Case avers that in some institutions big baskets of all kinds of lenses are placed before the boys, and they are ordered to choose any pair they please. Thus is the science of Donders and of great ophthalmologists made practical! Of those who could or did reply, 62 percent have no oculist and only 5 percent have even an optician; only 16 percent have any appropriation for such work whatsoever. The entire affair is a farce, an absurd bit of opera bouffe at best, that should be laughed and buried out of the sight of self-respecting, or of business-like people.

Wherever there was any attempt to go at the subject in the most amusingly inadequate way the results on demeanor, conduct, school, or hand work, promptly showed great improvement, the percentages of such improvement ranging from 15 to 54 percent, and averaging about 40 percent. The senior physician at Elmira, Dr. Frank L. Christian, writes: *"The general progress of the men, after receiving glasses was greatly improved, and far better results were obtained than previously."* And this, forget not, was after correction of manifest errors; without cycloplegia; for the purpose only of giving fair visual acuteness with one eye; muscular imbalance and astigmatism usually ignored; and differences of error of the two eyes (anisometropia) not considered. Even under these ruinous conditions Nature responds a little, and there is a degree of betterment gained. What would be the gain if accurate and thorough-going work were permitted,—if it were demanded! At the same time Government prides itself on being a democracy, the medical profession prides itself upon being scientific, and the people pride themselves on knowing how to conduct an enterprise in a businesslike manner!

1722 Walnut Street.

